# Social capital, the miniaturization of community, traditionalism and mortality: A population-based prospective cohort study in southern Sweden

**DOI:** 10.1016/j.ssmph.2021.100956

**Published:** 2021-11-06

**Authors:** Martin Lindström, Mirnabi Pirouzifard, Maria Rosvall

**Affiliations:** a Social Medicine and Health Policy, Department of Clinical Sciences and Centre for Primary Health Care Research, Lund University, S-205 02, Malmö, Sweden; b Department of Community Medicine and Public Health, Sahlgrenska Academy, Institute of Medicine, University of Gothenburg, Sweden

**Keywords:** Social capital, Cardiovascular diseases, Cancer, Mortality, Prospective cohort study, Survival analysis, Sweden

## Abstract

**Objectives:**

To investigate associations between social capital, miniaturization of community and traditionalism and all-cause, cardiovascular (CVD), cancer and other causes mortality.

**Study design:**

Prospective cohort study.

**Methods:**

The 2008 public health survey in Scania in the southernmost part of Sweden was conducted with a postal questionnaire posted to a stratified random sample aged 18–80. The response rate was 54.1%. The baseline survey was linked to 8.3-year prospective public death register data. Analyses were conducted with survival analyses, adjusting for relevant factors.

**Results:**

Among women 37.9% had low social participation and 37.8% low trust. Among men 40.9% had low social participation and 35.7% low trust. Low social capital (low social participation/low trust) and traditionalism (low social participation/high trust) have significantly higher total and cardiovascular mortality among women and men combined and among men, but not among women in the final models. The results for women are not significant in the full models for all-cause, CVD, cancer and all other causes mortality. Miniturization of community (high social participation/low trust) displays no statistically significant associations in the adjusted models. Social participation and trust, respectively, and total mortality show consistent Schoenfeld residuals over 8.3 years.

**Conclusions:**

The associations between low social capital, traditionalism and mortality are stronger for men than for women, and may be partly mediated by health-related behaviors.

## Highlights

• Combinations of cognitive (trust) and structural (social participation) social capital were analyzed.
• Traditionalism and low social capital are associated higher all-cause and CVD mortality among men.
• High social participation/low trust combination does not differ mortality from high social capital combination.
• It may be possible to analyze social capital using intersectional statistical aproaches.

## 1 Introduction

Social capital and health has been an important research area in public health since the first studies in the later part of the 1990s (Kawachi et al., 1997). Social capital was already early in this process suggested to affect health through pathways including psychosocial mechanisms, norms regarding health related behaviors, access to health care and other amenities, and as a protective factor against violent crime (Kawachi et al., 1999). Social capital has often been associated with lower mortality and better health (Lindström & Rosvall, 2019; Moore & Kawachi, 2017). Still, social capital in some social contexts and settings may instead be associated with poorer health outcomes, recently referred to as the dark side of social capital (Villalonga-Olives & Kawachi, 2017).

There are several theoretical approaches to the understanding of social capital. The cognitive or social cohesion approach originates from Coleman (1990) who defined social capital as reciprocity and trust, both horizontal trust in others between individual members of society with equal power and vertical trust in institutions across power gradients (Coleman, 1990). Putnam (1993; 2000) emphasized the importance of trust and reciprocity for the accomplishments of local and national governments, but also the importance of well-functioning social networks and social participation in society for trust and reciprocity. In fact, Putnam postulated a bi-directional relationship between social networks/social participation and trust/reciprocity, each enhancing the other (Putnam, 1993, 2000). The social network and social support approach to social capital originates from the French sociologist Pierre Bourdieu (Bourdieu, 1986; Portes, 1998). This pure social network approach regarding the study of social capital and health was proposed by Carpiano (Carpiano, 2006), and has been further developed by others (see e.g. Lin, 2017).

Current research on social capital and health goes beyond the separate social cohesion and social network approaches. Social capital and health research should include cognitive as well as structural dimensions in the analyses. Cognitive aspects of individuals, groups, networks and communities including trust and reciprocity should be combined with network aspects such as formal and informal network memberships and activities (Ehsan et al., 2019). Trust and reciprocity in tightly knit and narrowly defined social networks is defined as bonding social capital. In contrast, bridging social capital consists of trust and reciprocity with a wider scope, reaching across and between different social networks in society. Linking social capital connects individuals and groups across a power gradient (Moore & Kawachi, 2017). Second, social capital and health research should also define settings such as e.g. families, workplace, neighborhoods or communities (Moore & Kawachi, 2017). Third, studies on social capital and health should define level of analysis in terms of individual or group (aggregate or ecological) level (Islam et al., 2006; Macinko & Starfield, 2001). The present study combines cognitive and structural aspects of social capital by combining generalized trust in other people (horizontal trust) and social participation during the past year. Furthermore, our study conceptualizes the combination of cognitive and structural dimensions into categories such as miniaturization of community and traditionalism defined in the theoretical literature. Second, the setting of our study is the community in Scania in southern Sweden. Third, our level of analysis is the individual level.

The strength of weak social ties and norms, binding individuals within a wide array of organizations and networks and linking them over a wide range of formal, informal and institutional associational contexts and connections, has long been proposed as a strong prerequisite for common values (Granovetter, 1973) which may support and generate generalized trust in other people and reciprocity. In contrast, strong bonds within e.g. politically extreme or religiously fundamentalist social contexts may often not encourage generalized trust in other people and reciprocity in the entire society. This may also be the effect of one-issue movements, often with a top-down power structure. Still, it should be noted that moderate and traditional aspects of old religions provide a common ethical and moral basis that promote generalized trust in other people and reciprocity which would otherwise be difficult to achieve in an entirely secularized society (Lasch, 1995). Broad moderate political movements and other broad organizations in civil society may have the same effect (Lipset, 1995).

Fukuyama (1999) suggested that the decline in generalized trust in other people observed in the USA from the 1960s and onwards had produced a miniaturization of community resulting from a decreased radius of trust and sense of reciprocity. People often continue to participate in social activities, but within a decreased radius of trust in other people. According to Fukuyama, this process could be explained by e.g. trends of family dissolution and dissolution of common norms and values in an increasingly individualist, secularized and multicultural society (Fukuyama, 1999). Later, Putnam (2007) suggested that the trend towards increased cultural diversity in the USA had a lowering effect on generalized trust in other people by decreasing the radius of trust (Putnam, 2007). Woolcock and Narayan (2000) suggested that both bridging social activities, i.e. activities creating loose contact surfaces across social groups, and bonding social activities, i.e. activities strengthening already existing bonds within already existing and clearly defined social structures, are essential elements of social capital (Woolcock & Narayan, 2000). However, too much bonding social capital may affect this equilibrium by creating a condition of miniaturization of community, according to Fukuyama (Fukuyama, 1999). The opposite to miniaturization of community (high social participation/low generalized trust) we define as traditionalism (low social participation/high generalized trust) for two reasons. First, Putnam has suggested that the decrease in generalized trust in the USA since the 1960s is a cohort effect, i.e. older birth year cohorts with high trust have been replaced by new adult birth year cohorts with lower generalized trust (Putnam, 2000). Second, even in Sweden high generalized trust is more common among older than younger adults, while high social participation is more common among younger adults. Both these associations can be observed across clear age gradients going in opposite directions (Lindström, 2004).

The miniaturization of community has been observed to be positively associated with e.g. intermittent, non-daily smoking (but not with daily smoking) (Lindström, 2003), high alcohol consumption (Lindström, 2005a), consumption of illegally provided liquor (Lindström, 2005b) and poor psychological self-rated health (GHQ12) (Lindström, 2004) compared to the reference group with high social capital in both dimensions. However, the miniaturization of community population group did not have poorer self-rated health than the high social capital population segment with a combination of high social participation and high trust (Lindström, 2004) or higher hazard rate ratios of first time acute myocardial infarction (Ali et al., 2006) compared to the high social capital group within a 3-year follow-up.

No previous study has investigated the associations between the four categories high social capital, traditionalism, miniaturization of community and low social capital, and total, CVD, cancer and other causes mortality with a long follow-up period to our knowledge. The present study investigates these associations over a 8.3-year follow-up. Age, country of birth, socioeconomic status (by occupation and labor market status) were included in the multiple survival analyses as confounders, and chronic disease was included in order to control for reverse causation with special regard to social participation in social activities but also to some extent generalized trust in other people. Finally, the health-related behaviors leisure-time physical activity (Lindström & Rosvall, 2018), tobacco smoking and alcohol consumption were additionally included in the final multiple adjusted models in order to adjust for the health-related behavior pathway between social capital and mortality. The stability of the associations between social participation mortality and generalized trust in other people and all-cause mortality across the 8.3-year prospective follow-up period were also investigated by the analysis of proportionality tests and Schoenfeld residuals.

The aim of this study was to investigate associations between high social capital, traditionalism, miniaturization of community and low social capital, and all-cause (total), CVD, cancer and all other causes mortality using survival (Cox regression) analyses, adjusting for age, country of birth, socioeconomic status (SES), chronic disease, tobacco smoking, leisure-time physical activity and alcohol consumption, and stratifying for sex.

## 2 Methods

### 2.1 Study population

A public health survey was conducted in Scania (Skåne), the southernmost part of Sweden, in the autumn of 2008. This survey is based on a stratified random sample of the population aged 18–80 sampled from the official population register. A postal invitation letter including a questionnaire was sent, followed by three reminders to non-respondents. It was also possible to answer the questionnaire online. A total 28,198 persons responded, which resulted in a 54.1% participation rate. The study was conducted by Region Skåne which is the region responsible for the healthcare system in Skåne. The questionnaire includes items regarding sociodemographic circumstances, self-rated health, health-related behaviors, psychosocial conditions, social capital, and physical and psychosocial work conditions, and the questionnaire was conducted by Region Skåne, responsible for the healthcare system in Scania, to monitor public health in Scania in the autumn of 2008. The random sample was stratified according to municipality, city parts in the four major municipalities, age, sex and education in order to achieve sufficient statistical power to assess prevalence also in less populous municipalities and city parts of the four larger cities. The statistical power of the public health survey in 2008 is thus sufficient for the present study. The stratified sampling was conducted by statistics Sweden. Statistics Sweden also constructed a population weight that was used in all analyses in this study to compensate for the stratification in the sample. This baseline cross-sectional study from 2008 was linked to prospective mortality data from the National Board on Health and Welfare (*Socialstyrelsen*).

The present study, which is a prospective cohort study linking baseline public health survey data from 2008 to prospective mortality population register data was approved by the Ethical Committee (*Etikprövningsnämnden*) in Lund (No. 2010/343).

### 2.2 Dependent variable

Participants were followed from 27 August-14 November 2008 (depending on registration date of individual answer) until December 31, 2016 (8.3 years later), or until death. Totally 25,420 respondents were included in the present study. A total of 11,487 men and 13,933 women were included in the study, which excluded 2642 respondents with internally missing values on any of the included variables (social participation, generalized trust in other people, country of birth, socioeconomic status, self-reported chronic disease, smoking, alcohol consumption and leisure-time physical activity from the baseline postal questionnaire) in this study. The other 136 respondents of the original 28,198 respondents were lost to follow-up. All analyses were restricted to the 25,420 respondents with no internally missing values. The Swedish ten-digit person number system makes it possible to connect baseline data from the 2008 survey with the Swedish national cause of death register (the Swedish National Board on Health and Welfare) by a third party (private company). The ten-digit person numbers were deleted before delivery to researchers.

All-cause (total) mortality, cardiovascular mortality (I00–I98), cancer mortality (C00–C97), and all other cause mortality (other causes than I00–I98 and C00–C97) were analyzed in Table 2, Table 3. All-cause mortality is the sum the other three categories cardiovascular, cancer and all other cause mortality.

**Table 1:** Descriptive characteristics (%) of age (mean age), socioeconomic status (SES), country of birth, chronic disease, leisure-time physical activity, tobacco smoking and alcohol consumption. The 2008–2016 Public Health Survey of Scania, Sweden. Total population n = 25420.

	**Women** n = 13933 **Social participation and generalized trust combined**					**Men** n = 11487 **Social participation and generalized trust combined**				
	**High/high** n = 6091 41.8%	**Low/high** n = 2922 20.4%	**High/low** n = 257120.3%	**Low/low** n = 2349 17.5%	p-value	**High/high** n = 4797 41.1%	**Low/high** n = 2838 23.2%	**High/low** n = 1876 18.0%	**Low/low** n = 1976 17.7%	p-value
**Age**, yrs: mean ± SD ^a^	43.8 ± 14.6 (43.3–44.3)	51.7 ± 15.9 (50.9–52.5)	39.8 ± 16.5 (39.0–40.5)	47.5 ± 16.4 (46.6–48.4)	<0.001	44.3 ± 16.2 (43.7–44.8)	52.9 ± 16.9 (52.1–53.7)	40.2 ± 18.2 (39.3–41.1)	50.3 ± 17.4 (49.4–51.2)	<0.001
**Socioeconomic status**					<0.001					<0.001
Higher non-manual employees	12.9 (11.9–13.8)	4.6 (3.7–5.5)	5.9 (4.8–7.0)	2.0 (1.3–2.7)		17.3 (16.0–18.5)	5.4 (4.5–6.4)	6.4 (5.1–7.6)	2.7 (1.9–3.5)	
Medium non-manual employees	23.8 (22.5–24.0)	10.0 (8.7–11.2)	14.4 (12.9–16.0)	5.0 (3.9–6.1)		18.6 (17.3–20.0)	6.8 (5.7–7.8)	11.5 (9.8–13.2)	4.7 (3.7–5.8)	
Low non-manual employees	11.5 (10.5–12.5)	8.9 (7.7–10.1)	11.0 (9.5–12.5)	9.6 (8.1–11.2)		6.8 (5.9–7.7)	4.0 (3.1–4.9)	5.8 (4.5–7.1)	3.2 (2.3–4.2)	
Skilled blue-collar workers	7.9 (7.1–8.7)	9.2 (7.9–10.5)	12.4 (10.8–14.0)	9.4 (7.9–11.0)		10.7 (9.6–11.8)	12.2 (10.8–13.7)	14.8 (12.7–16.8)	10.4 (8.8–12.0)	
Unskilled blue-collar workers	9.5 (8.5–10.5)	14.1 (12.4–15.7)	13.4 (11.8–15.0)	16.1 (14.2–17.9)		9.6 (8.5–10.7)	12.7 (11.2–14.2)	15.4 (13.3–17.5)	18.1 (15.9–20.3)	
Self-employed/farmers	4.6 (4.0–5.3)	3.4 (2.6–4.2)	4.2 (3.2–5.1)	3.1 (2.2–4.0)		8.3 (7.4–9.2)	8.4 (7.1–9.7)	8.0 (6.5–9.5)	7.5 (6.1–8.9)	
	1.8 (1.4–2.2)	6.2 (5.1–7.3)	2.5 (1.8–3.2)	10.1 (8.5–11.7)		1.0 (0.6–1.3)	5.0 (4.0–6.0)	1.2 (0.7–1.7)	8.9 (7.1–10.6)	
Unemployed	2.1 (1.6–2.6)	5.1 (3.9–6.2)	4.7 (3.6–5.8)	7.4 (6.0–8.9)		1.9 (1.4–2.4)	3.9 (2.9–4.9)	4.3 (3.1–5.6)	7.4 (5.7–9.1)	
Students	9.4 (8.3–10.4)	5.7 (4.5–7.0)	14.1 (12.3–16.0)	9.5 (7.7–11.4)		6.8 (5.9–7.7)	4.9 (3.5–6.0)	11.8 (9.7–13.9)	6.0 (4.4–7.6)	
Old-age pensioners	12.1 (11.2–13.0)	28.2 (26.2–30.2)	10.0 (8.8–11.2)	19.6 (17.8–21.4)		13.2 (12.1–14.2)	29.9 (28.0–31.9)	10.2 (8.8–11.7)	23.3 (21.2–25.3)	
Long-term sick-leave	3.8 (3.1–4.4)	3.1 (2.2–4.1)	6.0 (4.9–7.3)	4.9 (3.7–6.2)		5.6 (4.7–6.5)	5.8 (4.4–7.2)	10.2 (8.2–12.1)	5.6 (4.2–6.9)	
Unclassified	0.6 (0.4–0.9)	1.5 (1.0–2.1)	1.2 (0.7–1.8)	3.2 (2.3–4.2)		0.3 (0.1–0.5)	1.2 (0.7–1.7)	0.4 (0.2–0.7)	2.2 (1.4–3.0)	
**Country of birth**	12.3 (11.1–13.4)	22.5 (20.4–24.7)	16.9 (14.8–19.0)	27.9 (25.4–30.4)	<0.001	11.9 (10.6–13.2)	19.7 (17.6–21.9)	17.6 (15.1–20.1)	26.8 (24.1–29.5)	<0.001
**Chronic disease**	24.0 (22.7–25.3)	32.6 (30.5–34.7)	29.0 (26.9–31.1)	40.8 (38.3–43.4)	<0.001	20.1 (18.8–21.5)	31.0 (29.0–33.1)	26.0 (23.5–28.5)	39.8 (37.0–42.6)	<0.001
**Leisure-time physical activity**	6.8 (6.0–7.5)	18.3 (16.5–20.1)	10.2 (8.7–11.7)	21.9 (19.8–24.1)	<0.001	8.2 (7.2–9.2)	21.1 (19.1–23.1)	12.3 (10.3–14.4)	27.2 (24.6–29.7)	<0.001
**Tobacco smoking**	10.0 (9.0–10.9)	17.6 (16.0–19.3)	15.4 (13.7–17.2)	25.5 (23.2–27.7)	<0.001	8.1 (7.1–9.1)	15.1 (13.3–16.8)	11.6 (9.7–13.4)	24.6 (22.1–27.2)	<0.001
**Alcohol consumption**					<0.001					<0.001
Never	5.9 (5.2–6.7)	22.7 (20.7–24.7)	10.2 (8.6–11.8)	27.3 (24.9–29.7)		4.0 (3.3–4.7)	12.7 (11.0–14.4)	6.4 (4.8–8.1)	18.3 (16.0–20.6)	
1 time per month or less	23.0 (21.7–24.3)	30.8 (28.7–33.0)	26.8 (24.7–28.9)	34.4 (32.0–36.8)		12.4 (11.2–13.6)	24.2 (22.2–26.2)	18.1 (15.8–20.4)	25.5 (23.1–27.9)	
2-4 times per month	40.0 (38.5–41.5)	27.8 (25.8–29.8)	39.7 (37.4–42.0)	23.3 (21.2–25.4)		41.8 (40.0–43.5)	31.7 (29.6–33.8)	43.5 (40.7–46.4)	28.5 (25.9–31.1)	
2-3 times per week	25.6 (24.3–26.9)	14.4 (12.9–15.9)	18.6 (16.8–20.4)	11.1 (9.6–12.7)		31.7 (30.1–33.3)	21.4 (19.6–23.1)	24.4 (22.0–26.7)	17.8 (15.8–19.9)	
4 times per week or more	5.5 (4.8–6.1)	4.3 (3.4–5.1)	4.7 (3.8–5.6)	3.9 (3.0–4.8)		10.1 (9.2–11.1)	10.1 (8.8–11.3)	7.6 (6.2–8.9)	9.9 (8.3–11.5)	
**Social participation**	0.0 (---)	100.0 (---)	0.0 (---)	100.0 (---)	<0.001	0.0 (--)	100.0 (---)	0.0 (---)	100.0 (---)	<0.001
**Generalized trust in other people**	0.0 (---)	0.00 (---)	100.0 (---)	100.0 (---)	<0.001	0.0 (--)	0.0 (---)	100.0 (---)	100.0 (---)	<0.001

^a^ p-value: Independent samples Anova-test, 2-tailed.

^b^ p-value: Pearson Chi Square test, 2-sided.

The values in parentheses are 95% confidence intervals for mean or percent based on bootstrap method with 2000 number of replicates.

**Table 2:** Hazard rate ratios (HRR:s) and 95% confidence intervals (95% CI:s) from Cox regression models for all-cause mortality and cause-specific mortality, showing association with combinations of social participation and generalized trust in other people. The 2008–2016 Scania public health survey with 8.3 years follow-up. Men and women combined. Total population n = 25420.

	Model 0		Model 1		Model 2		Model 3		Number of deaths
Cause of death	HR	(95%CI)	HR	(95%CI)	HR	(95%CI)	HR	(95%CI)	
**All causes** (Social participation/trust)									1304
High/high	1.0		1.0		1.0		1.0		
Low/high	3.4***	(2.9–4.0)	1.7***	(1.4–2.0)	1.6***	(1.3–1.9)	1.3**	(1.1–1.5)	
High/low	0.9	(0.7–1.2)	1.2	(0.9–1.5)	1.1	(0.8–1.4)	1.1	(0.8–1.3)	
Low/low	3.2***	(2.6–3.8)	2.1***	(1.8–2.6)	1.8***	(1.5–2.2)	1.4***	(1.2–1.7)	
**Cause-specific:**									
**Cardiovascular**									383
High/high	1.0		1.0		1.0		1.0		
Low/high	4.6***	(3.3–6.3)	2.1***	(1.5–2.9)	1.9***	(1.4–2.8)	1.5*	(1.1–2.2)	
High/low	0.7	(0.4–1.2)	0.8	(0.4–1.5)	0.8	(0.4–1.4)	0.7	(0.4–1.4)	
Low/low	4.1***	(2.9–5.9)	2.7***	(1.8–3.9)	2.3***	(1.5–3.3)	1.6*	(1.1–2.5)	
**Cancer**									517
High/high	1.0		1.0		1.0		1.0		
Low/high	2.6***	(2.0–3.3)	1.3*	(1.0–1.7)	1.3	(1.0–1.6)	1.2	(0.9–1.5)	
High/low	1.0	(0.7–1.4)	1.3	(0.9–1.7)	1.2	(0.9–1.7)	1.2	(0.9–1.7)	
Low/low	2.4***	(1.8–3.2)	1.6**	(1.2–2.2)	1.5**	(1.1–2.0)	1.3	(1.0–1.8)	
**Other causes**									404
High/high	1.0		1.0		1.0		1.0		
Low/high	3.8***	(2.7–5.2)	1.9***	(1.4–2.6)	1.6***	(1.2–2.3)	1.3	(0.9–1.8)	
High/low	1.1	(0.7–1.6)	1.3*	(0.8–2.0)	1.2	(0.8–1.8)	1.1	(0.7–1.7)	
Low/low	3.6***	(2.5–5.1)	2.4***	(1.7–3.5)	1.9**	(1.3–2.7)	1.4	(0.9–2.0)	

Model 0 Unadjusted.

Model 1 Adjusted for sex and age.

Model 2 Additionally adjusted for socioeconomic status, country of birth and chronic disease.

Model 3 Additionally adjusted for leisure-time physical activity, tobacco smoking and alcohol consumption (including all variables in models 0–3).

Significance levels: *p < 0.05, **p < 0.01, ***p < 0.001.

Weighted Hazard Ratios. Bootstrap method (2000 replicates) for variation estimation.

**Table 3:** Hazard rate ratios (HRR:s) and 95% confidence intervals (95% CI:s) from Cox regression models for all-cause mortality and cause-specific mortality, showing association with combinations of social participation and generalized trust in other people. The 2008–2016 Scania public health survey with 8.3 years follow-up. Stratified by gender. Total population n = 25420.

	Model 0		Model 1		Model 2		Model 3		Number of deaths
Cause of death	HR	(95%CI)	HR	(95%CI)	HR	(95%CI)	HR	(95%CI)	
**All causes** (High social participation/trust)									
**Women**									528
High/high	1.0		1.0		1.0		1.0		
Low/high	3.4***	(2.6–4.4)	1.7***	(1.3–2.2)	1.5**	(1.2–2.0)	1.2	(0.9–1.6)	
High/low	1.0	(0.7–1.5)	1.2	(0.8–1.8)	1.1	(0.8–1.7)	1.1	(0.7–1.6)	
Low/low	2.7***	(2.0–3.6)	1.9***	(1.4–2.6)	1.6**	(1.2–2.2)	1.2	(0.9–1.6)	
**Men**									776
High/high	1.0		1.0		1.0		1.0		
Low/high	3.4***	(2.7–4.2)	1.7***	(1.4–2.1)	1.6***	(1.3–2.0)	1.4**	(1.1–1.7)	
High/low	0.9	(0.7–1.3)	1.1	(0.8–1.5)	1.0	(0.8–1.4)	1.0	(0.7–1.4)	
Low/low	3.5***	(2.7–4.4)	2.3***	(1.8–2.9)	1.9***	(1.5–2.5)	1.6***	(1.2–2.0)	
**Cause-specific:**									
**Cardiovascular**									
**Women**									120
High/high	1.0		1.0		1.0		1.0		
Low/high	4.1***	(2.2–7.6)	1.8	(1.0–3.4)	1.6	(0.8–3.1)	1.1	(0.5–2.1)	
High/low	1.1	(0.2–4.5)	1.3	(0.3–5.8)	1.2	(0.3–5.3)	1.1	(0.3–5.0)	
Low/low	3.4***	(1.8–6.7)	2.3*	(1.2–4.5)	1.9	(0.9–3.9)	1.2	(0.5–2.5)	
**Men**									263
High/high	1.0		1.0		1.0		1.0		
Low/high	4.6***	(3.1–6.9)	2.2***	(1.5–3.4)	2.1***	(1.4–3.2)	1.7*	(1.1–2.7)	
High/low	0.5*	(0.2–1.0)	0.6	(0.3–1.2)	0.5	(0.3–1.1)	0.5	(0.3–1.1)	
Low/low	4.4***	(2.9–6.9)	2.8***	(1.8–4.4)	2.4***	(1.6–3.8)	1.9**	(1.2–3.0)	
**Cancer**									
**Women**									
High/high	1.0		1.0		1.0		1.0		243
Low/high	2.3***	(1.6–3.5)	1.2	(0.8–1.9)	1.2	(0.8–1.8)	1.0	(0.7–1.6)	
High/low	0.8	(0.5–1.4)	1.0	(0.6–1.7)	1.0	(0.6–1.7)	1.0	(0.6–1.6)	
Low/low	2.0**	(1.3–3.1)	1.5	(1.0–2.3)	1.4	(0.9–2.1)	1.1	(0.7–1.7)	
**Men**									274
High/high	1.0		1.0		1.0		1.0		
Low/high	2.7***	(1.9–3.9)	1.4	(1.0–2.0)	1.3	(0.9–1.9)	1.3	(0.9–1.8)	
High/low	1.2	(0.8–2.0)	1.5	(0.9–2.4)	1.4	(0.9–2.3)	1.4	(0.9–2.3)	
Low/low	2.7***	(1.8–4.1)	1.8**	(1.2–2.7)	1.6*	(1.1–2.5)	1.5	(1.0–2.3)	
**Other causes**									
**Women**									165
High/high	1.0		1.0		1.0		1.0		
low/high	5.5***	(3.2–9.4)	2.6***	(1.5–4.5)	2.2**	(1.3–3.9)	1.7	(0.9–3.0)	
high/low	1.3	(0.6–2.7)	1.6	(0.8–3.4)	1.4	(0.7–3.1)	1.4	(0.7–2.9)	
low/low	3.8***	(2.1–6.8)	2.6***	(1.5–4.8)	2.0**	(1.1–3.7)	1.5	(0.8–2.8)	
**Men**									239
High/high	1.0		1.0		1.0		1.0		
low/high	3.0***	(1.9–4.5)	1.6*	(1.0–2.4)	1.4	(0.9–2.1)	1.1	(0.7–1.7)	
high/low	1.0	(0.6–1.7)	1.2	(0.7–2.0)	1.0	(0.6–1.8)	1.1	(0.6–1.8)	
low/low	3.5***	(2.2–5.5)	2.3***	(1.5–3.7)	1.8*	(1.1–2.9)	1.4	(0.8–2.2)	

Model 0 unadjusted.

Model 1 Adjusted for age.

Model 2 Additionally adjusted for socioeconomic status, country of birth and chronic disease.

Model 3 Additionally adjusted for leisure-time physical activity, tobacco smoking and alcohol consumption (including all variables in models 0–3).

Significance levels: *p < 0.05, **p < 0.01, ***p < 0.001.

Weighted Hazard Ratios. Bootstrap method (2000 replicates) for variation estimation.

### 2.3 Independent variables

**Fig. 1:**
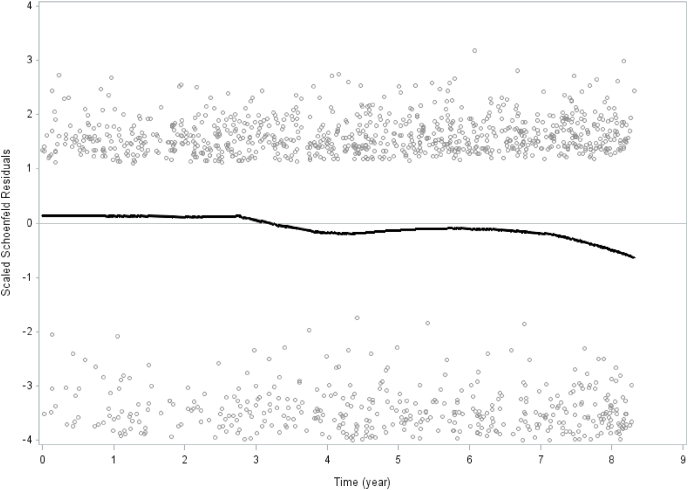
Schoenfeld residuals for social participation and all-cause mortality. Men and women combined. The 2008–2016 Public Health Survey of Scania, Sweden. Total population n = 25420.

**Fig. 2:**
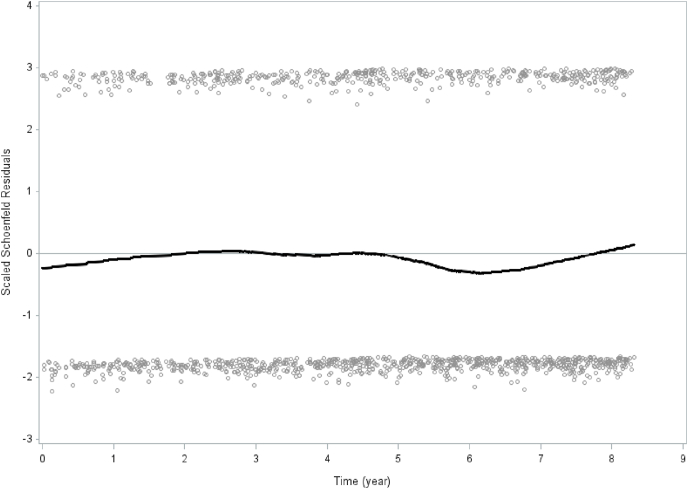
Schoenfeld residuals for generalized trust in other people and all-cause mortality. Men and women combined. The 2008–2016 Public Health Survey of Scania, Sweden. Total population n = 25420.

Analyses in Table 1, Table 3, Table 4 as well as Fig. 1, Fig. 2 were stratified by *sex*, while analyses presented in Table 2 were aggregated and instead adjusted for *sex*.

**Table 4:** HRs from Cox regression models for all-cause mortality, showing association with number of social participation activity sub-items. The 2008–2016 Scania public health survey with 8.3 years follow-up.

Social participation	Model 0		Model 1		Model 2		Model 3		Number of deaths
	**HR**	**(95%CI)**	**HR**	**(95%CI)**	**HR**	**(95%CI)**	**HR**	**(95%CI)**	
**All causes**									
0 activities	1.00		1.00		1.00		1.00		1304
1	0.78*	(0.62–0.99)	0.77*	(0.61–0.97)	0.84	(0.67–1.07)	0.91	(0.71–1.15)	
2	0.50***	(0.39–0.63)	0.62***	(0.49–0.78)	0.68**	(0.54–0.86)	0.77*	(0.61–0.98)	
3	0.36***	(0.28–0.46)	0.53***	(0.42–0.68)	0.60**	(0.47–0.77)	0.74*	(0.57–0.95)	
4	0.23***	(0.18–0.29)	0.40***	(0.32–0.51)	0.46***	(0.36–0.59)	0.59***	(0.45–0.76)	
5	0.19***	(0.14–0.25)	0.42***	(0.32–0.55)	0.49***	(0.38–0.65)	0.65**	(0.49–0.87)	
6	0.16***	(0.12–0.22)	0.40***	(0.29–0.55)	0.47***	(0.34–0.65)	0.65*	(0.46–0.91)	
7	0.12***	(0.08–0.18)	0.32***	(0.21–0.49)	0.39**	(0.26–0.59)	0.54**	(0.35–0.84)	
8	0.06***	(0.03–0.12)	0.21***	(0.10–0.43)	0.27***	(0.13–0.55)	0.38**	(0.18–0.78)	
9–14	0.02***	(0.00–0.22)	0.11*	(0.01–0.97)	0.12	(0.01–1.15)	0.18	(0.02–1.66)	

	n (%)	95% CI
0	1265 (5.0)	4.7–5.3
1	2096 (8.3)	7.9–8.7
2	2923 (11.3)	10.8–11.8
3	3801 (14.9)	14.4–15.4
4	4404 (17.3)	16.7–17.8
5	4052 (16.3)	15.8–16.9
6	3118 (12.2)	11.7–12.7
7	1972 (7.8)	7.4–8.2
8	1091 (4.3)	4.0–4.6
9–14	698 (2.7)	2.5–3.0

Model 0 Unadjusted.

Model 1 Adjusted for age and gender.

Model 2 Additionally adjusted for socioeconomic status, country of birth and chronic disease.

Model 3 Additionally adjusted for leisure-time physical activity, tobacco smoking and alcohol consumption.

Significance levels: *p < 0.05, **p < 0.01, ***p < 0.001.

Weighted Hazard Ratios. Bootstrap method (2000 replicates) for variation estimation.

Table of number of social participation activities (n = 25420).

The values in parentheses are 95% confidence intervals for percent based on bootstrap method with 2000 number of replicates.

*Age* is used as a continuous variable in the multiple survival analyses.

*Country of birth* is defined as either born in Sweden or born in another country.

*Socioeconomic status (SES)* includes non-manual employees in higher, medium and lower positions, skilled and unskilled manual workers, and self-employed/farmers in the active work force. The categories outside the active workforce include the unemployed, students, early retired before age 65, respondents on long-term sick leave, pensioners aged 65 or above, and unclassified.

*Chronic disease* was measured with the question “Do you have any long-term disease, ailment or injury, any disability or other weakness?”, with the alternative answers “Yes” and “No”.

*Tobacco smoking* was assessed with the question “Do you smoke?” which includes the alternatives “Yes, daily”, “Yes, but not daily” and “No”. The two first alternatives were collapsed making the variable dichotomous “smoking” versus “non-smoking”.

*Leisure-time physical activity* was assessed with the question “How much have you been exercising physically during leisure time in the past twelve months?”. Four response alternatives included 1) regular exercise (e.g. running, swimming, playing tennis, playing badminton, conducting exercise gymnastics or similar on average at least three times a week at least 30 min per occasion), 2) moderate, regular exercise in leisure time 1–2 times per week at least 30 min per occasion running, swimming, tennis, badminton or other leisure activity that makes you sweat, 3) moderate leisure time exercise walking, bicycling at least 2 h per week without sweating, and 4) sedentary leisure-time (walking, bicycling etc. less than 2 h per week). This variable was by dichotomized by collapsing the three first alternatives in order to achieve sedentary versus the others in Table 1.

*Alcohol consumption* was measured with the question “How often have you consumed alcohol during the past 12 months?” with the alternatives “4 times per week or more”, “2 to 3 times per week”, “2–4 times per month”, “1 time per month or less”, and “Never”.

*Social participation* in the past year measures the number of different activities in formal and informal groups in society: study circle/course at workplace, other study circle/course, union meeting, meeting of other organizations, theatre/cinema, arts exhibition, church, sports event, letter to the editor of a newspaper/journal, demonstration, night club/entertainment, large gathering of relatives, and private party. The item also included the alternative “None of the above”. The item was assessed as an index and it was dichotomized. Three alternatives or less during the past year depicts low social participation, four alternatives or more as high social participation. The social participation item has been used in Sweden since the 1960s/1970s (Hanson et al., 1997).

*Generalized trust in other people* is self-reported and reflects perception of trust. The statement “Generally, you can trust other people” with the four alternative answers “Do not agree at all”, “Do not agree”, “Agree”, and “Completely agree” was dichotomized with the two first alternatives depicting low trust and the two latter high trust.

Combining low/high social participation (dichotomized) and low/high trust (dichotomized) yielded the four categories high social participation/high trust (*high social capital*), low social participation/high trust (*traditionalism*), high social participation/low trust (*miniaturization of community*), low social participation/low trust (*low social capital*).

### 2.4 Statistics

Prevalence (%) of all variables by the four social participation/generalized trust categories were calculated stratified by sex. The differences among the four social participation/generalized trust categories were analyzed using ANOVA test for continuous variables and chi-square test for categorical variables (p-values) (Table 1). Hazard rate ratios (HRR:s) with 95% confidence intervals (95% CI:s) of all-cause, cardiovascular, cancer and other causes mortality according to the social participation/generalized trust categories were calculated for both sexes combined. Four models were calculated: model 0 unadjusted, model 1 adjusted for sex and age, model 2 adjusted for sex, age, country of birth, socioeconomic status, chronic disease, and model 3 additionally adjusted for tobacco smoking, leisure-time physical activity and alcohol consumption (Table 2). Hazard rate ratios (HRR:s) with 95% confidence intervals (95% CI:s) of all-cause, cardiovascular, cancer and other causes mortality according to the social participation/generalized trust categories were calculated stratified by sex. Four models were calculated also here: model 0 unadjusted, model 1 adjusted for age, model 2 adjusted for age, country of birth, socioeconomic status, and chronic disease, and model 3 additionally adjusted for tobacco smoking, leisure-time physical activity and alcohol consumption (Table 3). Associations between number of activities during the past year on the social participation index variable and mortality were analyzed in survival models with HRRs (95% CI) (Table 4). Follow-up days were measured from baseline to death or last follow-up date (2016-12-31), depending on which of the two that occurred first. Bootstrap analysis makes investigation of the sampling variability without distributional assumptions of the study population possible (SAS/STAT Software Survey Analysis, 2021). In order to ensure appropriate variance estimation on weighted data, bootstrap methods with 2000 numbers of replicates to obtain confidence intervals and p-values were used. Tests of proportionality for social participation and mortality and generalized trust and mortality, respectively, were conducted. The assumption of proportional hazards was determined by introducing an interaction term with time and social participation and generalized trust, respectively. Schoenfeld residuals were analyzed for social participation and mortality, and generalized trust and all-cause (total) mortality, respectively (Fig. 1, Fig. 2). In order to conduct calculations, the SAS software version 9.4 was used.

## 3 Results

Among women 37.9% reported low social participation and 37.8% low generalized trust. Among men 40.9% reported low social participation and 35.7% low generalized trust (adding low/high to low/low and high/low to low/low in top of Table 1, respectively).

Table 1 also conveys that among women some 41.8% had high social capital, 20.4% low social participation/high trust (traditionalists), 20.3% high social participation/ low trust (miniaturization of community) and 17.5% low social capital. Among men the proportions were 41.1% for high social capital, 23.2% traditionalists, 18.0% miniaturization of community and 17.7% low social capital. Differences between the four groups were significant for all variables. The highest mean age was observed for the traditionalist group (well above 50 years for both sexes) and the lowest for the miniaturization of community group (approximately 40 years for both sexes) among both men and women. The lowest proportion born abroad was observed in the high social capital group and the highest in the low social capital group for both sexes. High SES was most common in the high social capital group and low SES was most common in the low social capital group. The proportion with chronic disease was lowest in the high social capital category and lowest in the low social capital category. The proportion of smokers was 10.0% in the high social capital, 17.6% in the traditionalist, 15.4% in the miniaturization of community and 25.5% in the low social capital groups among women. The proportion of smokers was 8.1% in the high social capital, 15.1% in the traditionalist, 11.6% in the miniaturization of community and 24.6% in the low social capital groups among men. The proportion with low leisure-time physical activity was 6.8% in the high social capital, 18.3% in the traditionalist, 10.2% in the miniaturization of community and 21.9% in the low social capital groups among women. Similarly, the proportion with low leisure-time physical activity was 8.2% in the high social capital, 21.1% in the traditionalist, 12.3% in the miniaturization of community and 27.2% in the low social capital groups among men. Frequent alcohol consumption four times a week or more was most common in the low social capital group and least common in the high social capital group.

Table 2 shows that the traditionalist (low social participation/high trust) and low social capital categories consistently had higher HRR:s of all-cause mortality than the high social capital reference category throughout the analyses in models 0–3. The HRR for traditionalist men and women (combined) was 1.3 (1.1–1.5) in the fully adjusted final model 3, and the HRR for low social capital men and women (combined) was 1.4 (1.2–1.7) in model 3. In the cause-specific analyses of men and women combined, the HRR:s for the low social capital category were statistically significant in models 0–3 even after final additional adjustment for health-related behaviors in model 3. The miniaturization of community category did not have any statistically significant HRR:s for all-cause, cardiovascular, cancer and other causes mortality in any of the models 0–3. The HRR:s of cardiovascular mortality for the traditionalist category remained significant in models 0–3, but was reduced from 1.9 (1.4–2.8) in model 2 to 1.5 (1.1–2.2) in model 3. The HRR of cancer mortality for traditionalists was only statistically significant in models 0–1, and the HRR:s for other causes mortality was only statistically significant in models 0–2, being reduced from 1.6 (1.2–2.3) in model 2 to 1.3 (0.9–1.8) in model 3.

Table 3 shows that HRR:s of all-cause mortality decreased from 1.5 (1.2–2.0) in model 2 to 1.2 (0.9–1.6) in model 3 for traditionalist women, and from 1.6 (1.2–2.2) in model 2 to 1.2 (0.9–1.6) in model 3 for women with low social capital. In contrast, HRR:s of all-cause mortality remained statistically significant although they decreased from 1.6 (1.3–2.0) in model 2 to 1.4 (1.1–1.7) in model 3 for traditionalist men, and from 1.9 (1.5–2.5) in model 2 to 1.6 (1.2–2.0) in model 3 for men with low social capital. Both HRR:s for men thus remained statistically significant even after final additional adjustment for leisure-time physical activity, smoking and alcohol consumption. For women, no statistically significant HRR:s were observed for the cause-specific groups cardiovascular and cancer mortality in models 2 (adjusting for age, socioeconomic status, country of birth and chronic disease) and models 3 (additionally adjusting for leisure-time physical activity, smoking and alcohol consumption). The HRR:s of other cause mortality for traditionalist women decreased from 2.2 (1.3–3.9) in model 2 to 1.7 (0.9–3.0) in model 3, and for women with low social capital from 2.0 (1.1–3.7) in model 2 to 1.5 (0.8–2.8) in model 3. In contrast, traditionalist men had HRR 2.1 (1.4–3.2) in model 2 for cardiovascular mortality, which decreased to HRR 1.7 (1.1–2.7) in model 3. Men with low social capital had HRR 2.4 (1.6–3.8) for CVD mortality in model 2, which decreased to 1.9 (1.2–3.0) in model 3. The HRR:s of cancer mortality decreased from 1.6 (1.1–2.5 in model 2 to 1.5 (1.0–2.3) in model 3. Finally, men with low social capital had HRR 1.8 (1.1–2.9) for other causes mortality in model 2, which decreased to 1.4 (0.8–2.2) in model 3 when additionally adjusting for the three health-related behaviors. The miniaturization of community category showed no statistically significant association with 8.3-year all-cause, cardiovascular, cancer or all other causes mortality for either men or women in Table 3. In contrast, cardiovascular mortality remained in the proximity of HRR 0.5 for the miniaturization of community category among men throughout the analyses, although not statistically significant.

Table 4 shows that there is a clear all-cause mortality gradient following increasing number of activities reported on the social participation items during the past year. The higher the number of activities (of the 13 activities possible in the item) during the past year, the lower the HRR compared to the 0 activities during the past year reference group. The categories with 9–13 activities during the past year were collapsed into one category due to small numbers.

Test of proportionality (chi-Square test) between social participation and all-cause mortality showed p = 0.138 adjusted for age and sex, which indicates proportionality (p < 0.05 indicates lack of proportionality according to the test criteria). Test of proportionality (chi-Square test) between generalized trust in other people and all-cause mortality showed p = 0.469 adjusted for age and sex, which also indicates proportionality.

Schoenfeld residuals for social participation an and all-cause mortality (Fig. 1) as well as generalized trust and all-cause mortality (Fig. 2) show consistency and stability across time over the 8.3-year time period.

## 4 Discussion

Traditionalist (low social participation/high trust) and low social capital (low social participation/low trust) men and women combined had significantly higher all-cause (total) and cardiovascular mortality compared to the high social capital (high social participation/high trust) reference group throughout the multiple analyses. This pattern persisted for traditionalist and low social capital men but not women throughout the analyses stratified by sex. In contrast, the miniaturization of community group (high social participation/low trust) showed no significantly higher hazard rate ratios compared to the high social capital reference group throughout the multiple analyses for women and men combined as well as in the analyses stratified by sex. In contrast, for the miniaturization of community group among men the hazard rate ratios remained consistently approximately at 0.5 compared to the high social capital reference, on the verge of being statistically significant. The Schoenfeld residuals remained stable over 8.3 years with regard to the relationships between social participation and all-cause mortality as well as generalized trust in other people and all-cause mortality. The Schoenfeld residuals indicate the stability of social participation and mortality, and trust and mortality, respectively, in a general population over time.

The results of this study suggest that low social capital is significantly associated with higher all-cause mortality and cardiovascular mortality for women and men combined as well as among men. The results of this study with a 8.3-year follow-up support a previous prospective study with a shorter follow-up period (Lindström & Rosvall, 2019). Furthermore, the analyses of the Schoenfeld residuals for social participation and all-cause mortality, and trust and all-cause mortality, respectively, show almost constant effects across time over a 8.3-year follow-up period, which has not been previously analyzed. These findings partly contrast with the conclusions from a systematic review of prospective studies concerning social capital and mortality, which summarized its findings with the statement that evidence supporting the association between social capital and mortality was limited (Choi et al., 2014).

The miniaturization of community combination high social participation/low trust population group did not convey any statistically significant associations with mortality.

The hazard rate ratios for this category were sometimes even lower than for the high social capital group, and for cardiovascular mortality among men approximately 0.5 throughout the multiple analyses compared to the high social capital group of men in Table 3, although not statistically significant. Health-related behaviors such as intermittent non-daily tobacco smoking and consumption of homemade and smuggled liquor during the past year or even poor psychological health do not automatically result in increased mortality during a 8.3-year period. Instead, the miniaturization of community group should be more thoroughly scrutinized in future studies bearing this group in mind as a group of individualists in a postmodern society with most likely some protective characteristics calling for further investigation. Instead, the traditionalist combination low social participation/high trust segment conveyed statistically significant associations with all-cause and cardiovascular mortality among men and women combined as well as with all-cause and cardiovascular morality among men before adjustments for health-related behaviors. Health-related behaviors may causally mediate associations between especially low social participation and all-cause mortality and in particular cardiovascular mortality. These results conform to previous results that showed increased HRR:s of first-time acute myocardial infarction for the traditionalist population segment (Ali et al., 2006).

The analyses in the present study are based on the dichotomization of two items that represent two separate different aspects of social capital according to the literature. The present analyses thus follow the suggestion that cognitive and network aspects of social capital should be combined in the study of social capital and health (Ehsan et al., 2019). This approach based on two dichotomizations is methodologically still somewhat crude and may plausibly be improved. One direction of improvement may be to apply intersectional analysis of individual heterogeneity and discriminatory accuracy (AIHDA) (Axelsson Fisk et al., 2021) and multilevel analysis of individual heterogeneity and discriminatory accuracy (Axelsson Fisk et al., 2018) to a more complex analysis of intersections of aspects of social capital including generalized trust in other people, reciprocity and social participation. The analyses of highly correlated items such as different aspects of generalized trust should be avoided in such analyses, but this partly new methodology still opens up the possibility to analyze several dimensions of social capital with the additional inclusion of demographic characteristics.

### 4.1 Strengths and limitations

This study is population-based, longitudinal, prospective and comparatively large.

The 54.1% participation rate is moderate in the western and Swedish context of 2008. However, the respondents are acceptably representative of the general population in Scania (Skåne) at that point in time. Some underrepresentation of young people, men, born abroad and those with low socioeconomic status was observed. The risk of selection bias is thus comparatively small, which has been previously discussed (Lindström et al., 2014; Lindström & Rosvall, 2019).

The causal links between social capital and chronic disease mortality entail psychosocial stress, norms regarding health related behaviors, access to healthcare and other amenities, and protection from violent crime (Kawachi et al., 1999). It may be argued that an 8.3-year follow-up may be regarded as a rather short time-span to determine CVD and cancer. However, social capital and mortality and chronic disease mortality have been studied in prospective studies with follow-up time ranging from 2 to 35 years (Choi et al., 2014). Furthermore, both social capital in the form of social participation and generalized trust in other people, and health-related behaviors such as smoking, leisure-time physical activity and alcohol consumption are comparatively stable over time. In this study, the Scoenfeld residuals for social participation and generalized trust in other people were proportional (in relation to all-cause mortality) over 8.3 years.

A previous study of social capital and 5.3-year mortality has shown that mortality in the 18–34 age range is 100 times lower than in the 65–80 age range. However, associations between the four social capital variables (in that study) social participation and generalized trust in other people were similar across age ranges (Lindström & Rosvall, 2019). Furthermore, mortality across age ranges in the population sample are similar to mortality across age ranges in the general population in Sweden during the same time-period (Heimersson, 2013; Lindström & Rosvall, 2019).

The social participation item and its dichotomization between three and four alternatives has been used in Sweden since the 1970s. The validity of the social participation item is high (Hanson et al., 1997). The generalized trust item is generally used internationally (Putnam, 2000). The fact that the social participation item does not measure intensity/number of occasions for each activity may be seen as a weakness. Socioeconomic status (SES) was measured according to occupation and employment status on the labor market in this study. Other SES dimensions include income and education. The three dimensions occupation, income and education are generally considered as highly correlated but not identical. Income is not included in the public health questionnaire in Skåne 2008. The inclusion of education in the analyses does not alter the results, it only decreases the number of participants included in the analyses due to a higher number of internally missing for the education variable compared to the occupation variable. There is thus no reason to suspect residual confounding due to the omission of the education variable from the survival analyses. The leisure-time physical activity item has acceptable validity and reliability in relation to golden standard measures that assess four-day heart rate monitoring and whole-day calorimetry and double-labelled water (Wareham et al., 2003). The smoking item has high validity and reliability (Wells et al., 1998). Official population register data in Sweden generally have high validity. The broad cardiovascular, cancer and other causes groups of diagnoses are very broad and given the high validity of Swedish register data the risk of misclassification is very small.

There are framework limitations connected with the theoretical framework used in this manuscript, and we acknowledge that the quantitative methodology deployed in this research carries the risk of reproducing ideological assumptions surrounding the contested concepts of community (Joseph, 2002), tradition (Adorno, 1992), trust (Withers, 2018), social capital (Pawar, 2006), and gender (Simandan, 2019).

### 4.2 Conclusions

The associations between traditionalism as well as low social capital and all-cause and cardiovascular mortality are stronger for men than for women, and may be partly mediated by health-related behaviors. Miniturization of community (high social participation/low trust) displays no statistically significant associations in the adjusted models. Complex analyses combining several dimensions and traits of social capital may be conducted in the future using AIHDA/MAIHDA statistical techniques.

## Ethical approval

The present study was approved by the Ethical Committee (*Etikprövningsnämnden*) in Lund (No. 2010/343).

## Declaration of competing interest

None declared.

## References

[bib1] Adorno T.W. (1992). On tradition. Telos.

[bib2] Ali S.M., Merlo J., Rosvall M., Lithman T., Lindström M. (2006). Social capital, the miniaturisation of community, traditionalism and first time acute myocardial infarction: A prospective cohort study in southern Sweden. Social Science & Medicine.

[bib3] Axelsson Fisk S., Lindström M., Perez-Vicente R., Merlo J. (2021). Understanding the complexity of socioeconomic disparities in smoking prevalence in Sweden: A cross-sectional study applying intersectionality theory. BMJ Open.

[bib4] Axelsson Fisk S., Mulinari S., Wemrell M., Lekie G., Pereez-Vicente R., Merlo J. (2018). Chronic obstructive lung disease in Sweden: An intersectional multilevel analysis of individual heterogeneity and discriminatory accuracy. SSM Population Health.

[bib5] Bourdieu P., Richardson J. (1986). Handbook of theory and research for the sociology of education.

[bib6] Carpiano R.M. (2006). Towards a neighborhood resource-based theory of social capital for health: Can Bourdieu and sociology help?. Social Science & Medicine.

[bib7] Choi M., Mesa-Frias M., Nuesch E., Hargreaves J., Prieto-Merino D., Bowling A., Davey Smith G., Ebrahim S., Dale C., Casas J.P. (2014). Social capital, mortality, cardiovascular events and cancer: A systematic review of prospective studies. International Journal of Epidemiology.

[bib8] Coleman J.S. (1990).

[bib9] Ehsan A., Klaas H.S., Bastianen A., Spini D. (2019). Social capital and health: A systematic review of systematic reviews. SSM Population Health.

[bib10] Fukuyama F. (1999). Human nature and the reconstitution of social order.

[bib11] Granovetter M. (1973). The strength of weak ties. American Journal of Sociology.

[bib12] Hanson B.S., Östergren P.O., Elmståhl S., Isacsson S.O., Ranstam J. (1997). Reliability and validity assessments of of measures of social network, social support and control- results from the Malmö Shoulder and Neck Study. Scandinavian Journal of Social Medicine.

[bib13] Heimersson I. (2013).

[bib14] Islam M.K., Merlo J., Kawachi I., Lindström M., Gerdtham U.G. (2006). Does egalitarianism matter? A literature review. International Journal for Equity in Health.

[bib15] Joseph M. (2002).

[bib16] Kawachi I., Kennedy B.P., Glass R. (1999). Social capital and self-rated health: A contextual analysis. American Journal of Public Health.

[bib17] Kawachi I., Kennedy B.P., Lochner K., Prothrow-Stith D. (1997). Social capital, income inequality, and mortality. American Journal of Public Health.

[bib18] Lasch C. (1995). The revolt of the elites- and the betrayal of democracy.

[bib19] Lin N. (2017). Social capital.

[bib20] Lindström M. (2003). Social capital and the miniaturization of community among daily and intermittent smokers: A population-based study. Preventive Medicine.

[bib21] Lindström M. (2004). Social capital, the miniaturization of community and self reported global and psychological health. Social Science & Medicine.

[bib22] Lindström M. (2005). Social capital, the miniaturization of community and high alcohol consumption: A population-based study. Alcohol and Alcoholism.

[bib23] Lindström M. (2005). Social capital, the miniaturization of community and consumption of home made liquor and smuggled liquor during the past year: A population-based study. The European Journal of Public Health.

[bib24] Lindström M., Fridh M., Rosvall M. (2014). Economic stress in childhood and adulthood, and poor psychological health: Three life course hypotheses. Psychiatry Research.

[bib25] Lindström M., Rosvall M. (2018). Economic stress and low leisure-time physical activity: Two life-course hypotheses. SSM Population Health.

[bib26] Lindström M., Rosvall M. (2019). Two theoretical strands of social capital, and total, cardiovascular, cancer and other mortality: A population-based prospective cohort study. SSM Population Health.

[bib27] Lipset S.M. (1995).

[bib28] Macinko J., Starfield B. (2001). The utility of social capital in research on health determinants. The Milbank Quarterly.

[bib29] Moore S., Kawachi I. (2017). Twenty years of social capital and health research A glossary. Journal of Epidemiology & Community Health.

[bib30] Pawar M. (2006). Social””capital. The Social Science Journal.

[bib31] Portes A. (1998). Social capital: Its origins and applications in modern sociology. Annual Review of Sociology.

[bib32] Putnam R.D. (1993).

[bib33] Putnam R.D. (2000).

[bib34] Putnam R.D. (2007). E pluribus unum: Diversity and community in the twenty-first century. The 2006 johan skytte prize lecture. Scandinavian Political Studies.

[bib501] (2021). SAS/STAT software survey analysis.

[bib35] Simandan D. (2019). Revisiting positionality and the thesis of situated knowledge. Dialogues in Human Geography.

[bib36] Villalonga-Olives E., Kawachi I. (2017). The dark side of social capital: A systematic review of the negative health effects of social capital. Social Science & Medicine.

[bib37] Wareham N.J., Jakes R.W., Rennie K.L., Schuit J., Mitchell J., Hennings S., Day N.E. (2003). Validity and repeatability of a simple index derived from the short physical activity questionnaire used in the European Prospective Investigation into Cancer and Nutrition (EOIC) study. Public Health Nutrition.

[bib38] Wells A.J., English P.B., Posner S.F., Wagenknecht L.E., Perez-Stable E.J. (1998). Classification rates for current smokers misclassified as non-smokers. American Journal of Public Health.

[bib39] Withers C.W. (2018). Trust-in geography. Progress in Human Geography.

[bib40] Woolcock M., Narayan S. (2000). Social capital: Implications for development theory, research and policy. The World Bank Research Observer.

