# Supplementary material for: Social capital, the miniaturization of community, traditionalism and mortality: A population-based prospective cohort study in southern Sweden
Source: SSM Popul Health. 2021 Nov 6;16:100956. doi: 10.1016/j.ssmph.2021.100956 (PMC8591417; doi:10.1016/j.ssmph.2021.100956)
Supplement: Multimedia component 1 [file mmc1.docx]

| **Table S1.** HRs from logistic regression models for all-cause mortality, showing association with social participation and generalized trust in other people. The 2008 -2016 Scania public health survey with 8.3 years follow-up. Men and women combined. Total population n=25420. | | | | | | | | | |
| --- | --- | --- | --- | --- | --- | --- | --- | --- | --- |
| **Cause of death (All causes)** | | | | | | | | | |
|  | **Model 0** | | | **Model 1** | | **Model 2** | | **Model 3** | |
| **Variable** | REF | OR | (95%CI) | OR | (95%CI) | OR | (95%CI) | OR | (95%CI) |
| Social participation | 0 | **3.5***** | (3.0-4.0) | **1.8***** | (1.6-2.1) | **1.6***** | (1.4-1.9) | **1.3**** | (1.1-1.6) |
| Generalized trust in other people | 0 | 0.9 | (0.8-1.1) | 1.3 | (1.1-1.5) | 1.2 | (1.0-1.3) | 1.1 | (0.9-1.3) |
| Model 0 Unadjusted. Model 1 Adjusted for sex and age. Model 2 Additionally adjusted for socioeconomic status, country of birth and chronic disease. Model 3 Additionally adjusted for leisure-time physical activity, tobacco smoking and alcohol consumption (including all variables in models 0-3). Significance levels: * p<0.05, ** p<0.01, *** p<0.001. Weighted Odds Ratios. Bootstrap method (2000 replicates) for variation estimation. | | | | | | | | | |

| **Table S2.** HRs from logistic regression models for all-cause mortality, showing association with social participation, Generalized trust in other people and interaction between them. The 2008 -2016 Scania public health survey with 8.3 years follow-up. Men and women combined. Total population n=25420. | | | | | | | | | | | | |
| --- | --- | --- | --- | --- | --- | --- | --- | --- | --- | --- | --- | --- |
| **Cause of death (All causes)** | | | | | | | | | | | | |
|  | **Model 0** | | | **Model 1** | | | **Model 2** | | | **Model 3** |  | |
| **Variable** | Coefficient | Standard Error | Exp (Coeff) | Coefficient | Standard Error | Exp (Coeff) | Coefficient | Standard Error | Exp (Coeff) | Coefficient | Standard Error | Exp (Coeff) |
| Social participation | 1.26 | 0.09 | **3.53***** | 0.57 | 0.09 | **1.78***** | 0.47 | 0.10 | **1.60***** | 0.26 | 0.10 | **1.30**** |
| Generalized trust in other people | -0.06 | 0.12 | 0.94 | 0.17 | 0.13 | 1.19 | 0.09 | 0.13 | 1.10 | 0.06 | 0.13 | 1.06 |
| Social participation* Generalized trust in other people | -0.03 | 0.15 | 0.97 | 0.08 | 0.16 | 1.08 | 0.07 | 0.16 | 1.07 | 0.04 | 0.16 | 1.04 |
| Model 0 Unadjusted. Model 1 Adjusted for sex and age. Model 2 Additionally adjusted for socioeconomic status, country of birth and chronic disease. Model 3 Additionally adjusted for leisure-time physical activity, tobacco smoking and alcohol consumption (including all variables in models 0-3). Significance levels: * p<0.05, ** p<0.01, *** p<0.001. Weighted Odds Ratios. Bootstrap method (2000 replicates) for variation estimation. | | | | | | | | | | | | |
